# mRNA vaccine sequence and structure design and optimization: Advances and challenges

**DOI:** 10.1016/j.jbc.2024.108015

**Published:** 2024-11-26

**Authors:** Lei Jin, Yuanzhe Zhou, Sicheng Zhang, Shi-Jie Chen

**Affiliations:** 1 Department of Physics and Astronomy, University of Missouri, Columbia, Missouri, USA; 2 Department of Biochemistry, MU Institute for Data Science and Informatics, University of Missouri, Columbia, Missouri, USA

**Keywords:** mRNA vaccine, mRNA sequence design, vaccine design, machine-learning, RNA structure

## Abstract

Messenger RNA (mRNA) vaccines have emerged as a powerful tool against communicable diseases and cancers, as demonstrated by their huge success during the coronavirus disease 2019 (COVID-19) pandemic. Despite the outstanding achievements, mRNA vaccines still face challenges such as stringent storage requirements, insufficient antigen expression, and unexpected immune responses. Since the intrinsic properties of mRNA molecules significantly impact vaccine performance, optimizing mRNA design is crucial in preclinical development. In this review, we outline four key principles for optimal mRNA sequence design: enhancing ribosome loading and translation efficiency through untranslated region (UTR) optimization, improving translation efficiency *via* codon optimization, increasing structural stability by refining global RNA sequence and extending in-cell lifetime and expression fidelity by adjusting local RNA structures. We also explore recent advancements in computational models for designing and optimizing mRNA vaccine sequences following these principles. By integrating current mRNA knowledge, addressing challenges, and examining advanced computational methods, this review aims to promote the application of computational approaches in mRNA vaccine development and inspire novel solutions to existing obstacles.

Vaccination has been the most significant health intervention in reducing mortality since 1974 (1). The World Health Organization estimates that global vaccination has prevented 154 million deaths over the past 50 years (1). Vaccines are designed to train the immune system to recognize antigens produced by pathogens or malignant cells (2, 3, 4, 5) and stimulate a robust adaptive immune response against infections or cancers (6, 7). Recent advances in messenger RNA (mRNA) technology have enabled the delivery of antigen-encoding mRNA molecules into host cells, facilitating antigen expression within the human body (4). Compared to conventional vaccines (*e.g.*, inactivated, live-attenuated, and subunit/recombinant/polysaccharide/conjugate types), mRNA vaccines offer several distinct advantages. First, mRNA can be designed and manufactured in a rapid, scalable, and cost-effective manner owing to the high yields of cell-free *in vitro* transcription (IVT) technique (4, 8). For example, the severe acute respiratory syndrome coronavirus 2 (SARS-CoV-2) mRNA vaccine mRNA-1273 entered clinical testing just 2 months after the genetic sequence of SARS-CoV-2 was identified (9, 10). Second, the mRNA vaccine is free of infectious pathogens, eliminating the risk of infection upon vaccination (4). Additionally, the cell-free, *in vitro* manufacturing environment minimizes safety concerns typically associated with other platforms, such as cell-derived impurities and viral contaminants (11). Third, the mRNA vaccine can encode multiple specific antigens in a single formulation, allowing for both precise and robust immune responses against resilient pathogens (8, 12).

Despite the remarkable success of mRNA vaccines in coronavirus disease 2019 (COVID-19) prevention, several challenges remain for their broader applications, particularly in cancer treatment—the field that initially sparked their development (13, 14, 15). One significant hurdle is the insufficient antigen-expressing efficiency of artificial mRNAs used in vaccines, which often fails to elicit a long-lasting immune response, necessitating multiple booster injections to establish effective protection (16, 17). Another obstacle is the intrinsic thermal instability of mRNA molecules, requiring low-temperature storage and posing logistical challenges that can impede vaccine distribution during a pandemic (17). Furthermore, the reactogenicity^1^ of artificial mRNA and its delivery carriers, along with potential undesirable protein translation, may lead to unexpected side effects or even hypersensitivity reactions (18, 19, 20).

Advances in mRNA molecular and metabolic biology have led to potential solutions for some of the challenges in designing effective mRNA vaccines (21, 22). These approaches include optimizing mRNA sequences and structures to enhance ribosome loading and translation efficiency (TE), increasing mRNA thermal stability by designing sequences that fold into more stable structures, reducing unstructured RNA local motifs that may trigger cellular immune responses or attract in-cell ribonucleases, as well as avoiding slippery sequence motifs and downstream frameshift structural signals to maintain translation fidelity. These strategies collectively aim to improve the efficacy and stability of mRNA-based vaccines. Since the properties of an mRNA vaccine are largely determined by its primary sequence, most of the current efforts to improve efficacy focus on sequence design and optimization (21).

In eukaryotic cells, precursor mRNAs (pre-mRNAs) produced by transcription need to undergo processing to form mature mRNAs before they are ready for translation during protein synthesis. This maturation process typically involves three main steps: 5′ end capping, 3′ end polyadenylation, and intron splicing. In order to have an improved expression efficiency and translation fidelity, artificially designed mRNAs used for vaccines should ideally be mature and ready for translation before entering host cells (14, 15, 23). Since the IVT technique can synthesize RNA molecules from DNA templates in a controlled laboratory environment, it simplifies production and maturation of custom-designed mRNA sequences. This method retains only the regulatory elements essential for protein synthesis, allowing researchers to design, evaluate, and optimize the functions of each individual mRNA segment separately to achieve higher expression efficiency, in-solution/cell stability, and expression fidelity (21, 22, 24). The simplified mRNA produced by IVT consists of five components: 5′ cap, 5′ untranslated region (UTR), open reading frame (ORF), 3′ UTR, and 3′ end (*i.e.*, poly(A) tail). The IVT produced mRNA sequence starts with the 5′ cap, usually modified guanosine (7-methylguanosine, abbreviated m^7^G), whose purpose is to stabilize IVT mRNA against 5′ exonucleolytic degradation and initiate translation within the cell (25, 26, 27). Following the 5′ cap, the 5′ UTR contains crucial regulatory regions such as the ribosome binding site (RBS) and internal ribosome entry site (IRES). These elements control translation by recruiting the ribosome and other translation factors (28, 29, 30). The ORF, located between the start and stop codons, contains the essential coding sequence (CDS) that encodes the target protein antigen (31, 32, 33). Without the need of intron splicing in IVT-produced mRNA, the ORF can simply consist of a single continuous CDS (34). The 3′ UTR, together with the 3′ end, protects the mRNA from enzymatic degradation in the cytoplasm by recruiting poly(A)-binding proteins (PABPs) (29, 31, 35, 36). Figure 1 illustrates a simplified manufacturing procedure and mechanism of action for mRNA vaccines.

**Figure 1:**
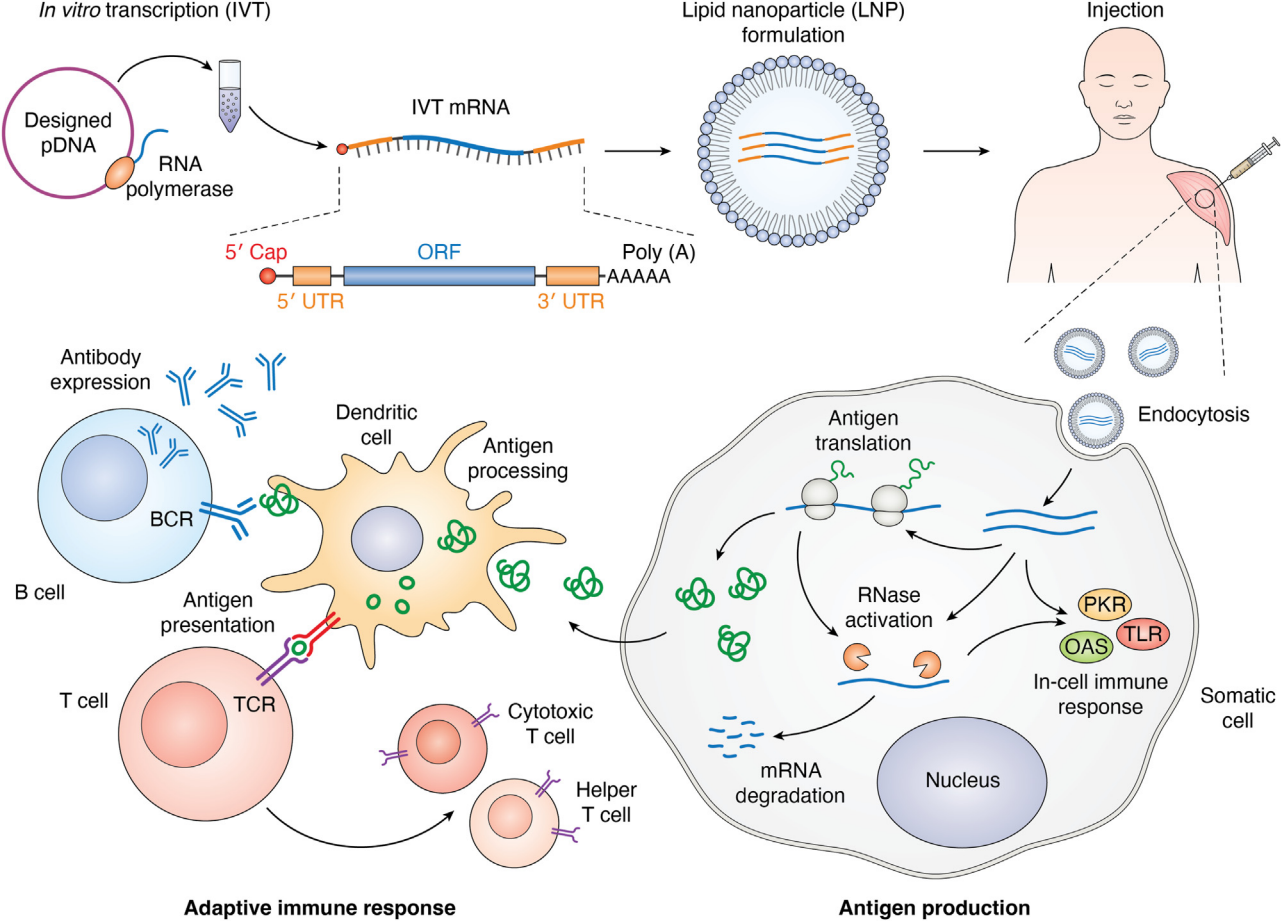
**Simplified mRNA vaccine manufacturing procedure and mechanism of action.** The artificial mRNAs, designed based on the antigen protein sequence, are *in vitro* transcribed and encapsulated in lipid nanoparticles (LNPs). Upon intramuscular injection, LNPs facilitate mRNAs’ entry into muscle cells *via* endocytosis. The translated antigens stimulate adaptive immune responses, activating B and T cells against potential infections. Injected mRNAs are subsequently degraded by cellular ribonucleases (RNases).

Most current computational models for designing and optimizing mRNA sequences focus on individual components of *in vitro* transcribed mRNAs (21). Over the past few years, the 5′ UTR—a major regulatory region of mRNA that determines ribosome loading efficiency—has been investigated (37, 38). Based on these findings, computational models have been proposed to design artificial mRNAs by screening/selecting naturally occurring 5′ UTRs. Additionally, some of the models are also capable of further optimizing/designing 5′ UTR sequences. Meanwhile, several studies have investigated the ORF region containing the CDS (31, 32, 33). However, the high variability in codons makes exhausting all possible CDS configurations computationally infeasible. For instance, an ORF encoding a target antigen like the SARS-CoV-2 spike protein could theoretically have over 10^632^ possible CDS variations due to codon diversity (23). While current clinically available mRNA vaccines generally select the optimal codon for each amino acid (23), there remains considerable room for improvement in ORF design when considering other ORF-related factors that impact the vaccine’s overall performance. Even if one can identify the optimal sequence for each individual mRNA component, these separately designed segments require validation when integrated into a full mRNA sequence. This step is crucial because long-range interactions between segments and global refolding of the mRNA may lead to unexpected outcomes (39, 40, 41).

In this review, we introduce currently available computational approaches for mRNA sequence design and optimization together with their applications in mRNA vaccine development. By addressing challenges in mRNA vaccine development, and exploring advanced computational approaches, we aim to promote the development of computational methods in mRNA vaccine design and inspire new strategies to overcome existing challenges.

## Computational mRNA vaccine sequence and structure design and optimization

**Figure 2:**
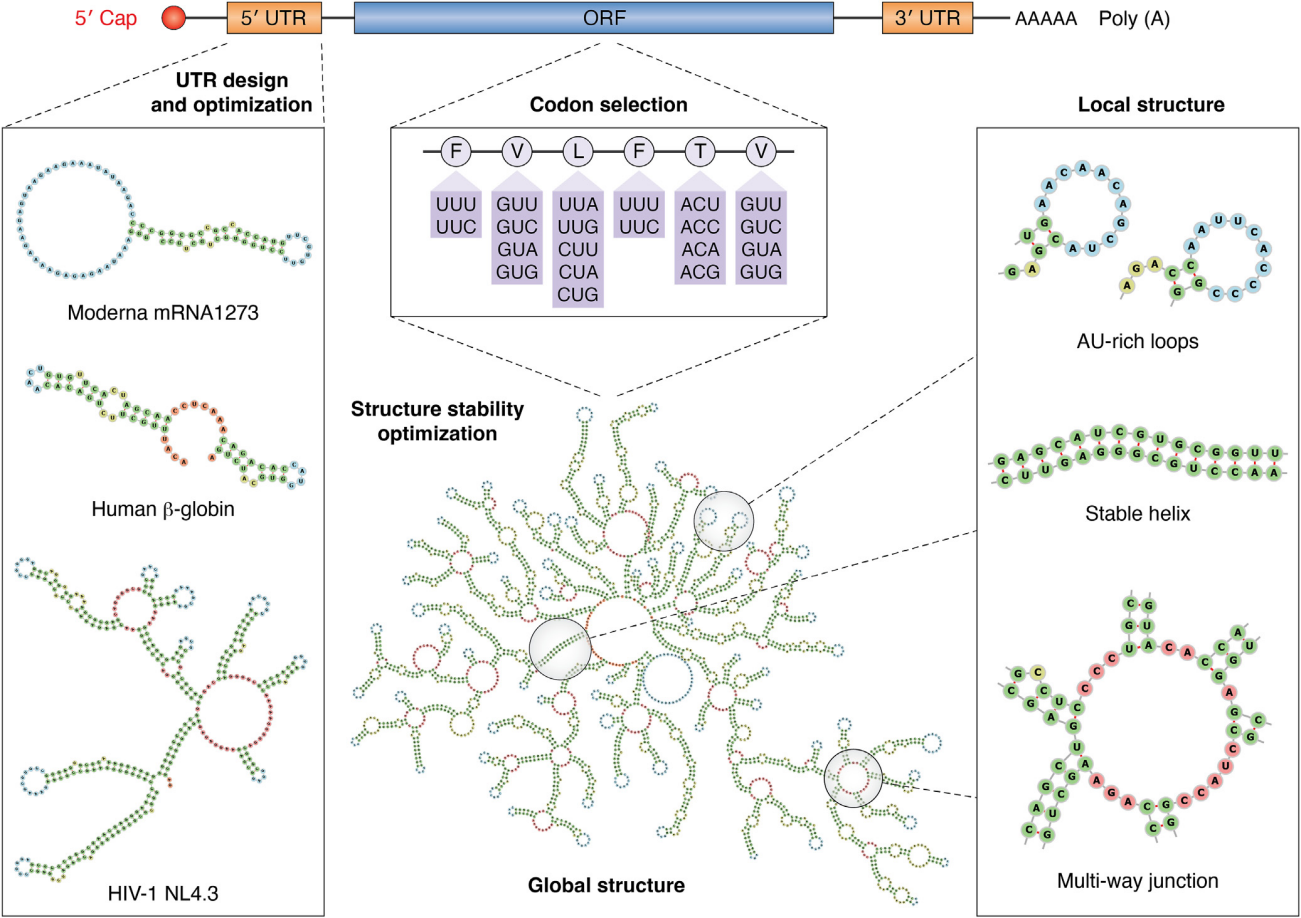
**Illustration of *in vitro* transcription (IVT) mRNA components and their corresponding design/optimization processes:** 1. Designing and optimizing untranslated region (UTR) sequence; 2. Optimizing open reading frame (ORF) sequence with optimal codon usage; 3. Adjusting and fine-tuning global and local structures. The illustrated 2D structures are generated using RNAfold (112), RNA structure (72), Vfold2D (114), and a landscape-zooming cotranscriptional folding model (183).

To maximize the potential of mRNA vaccines, an optimally designed IVT mRNA sequence should achieve high antigen expression efficiency, exhibit low degradation rates both in solution and within cells, and minimize reactogenicity. Based on the functional segmentation of the mRNA sequence, the design objectives for the UTR, ORF, and the overall sequence can be classified into four key principles (see Fig. 2 for an illustration of the IVT mRNA components and their corresponding design/optimization processes).

1. Design and optimize UTRs to enhance ribosome loading and translation efficiency.
2. Optimize codon usage in the ORF to improve translation efficiency.
3. Refine the global sequence to adopt a stable structure for increased in-solution stability.
4. Adjust local sequences to avoid specific sequence/structure motifs, thereby extending in-cell lifetime and improving expression fidelity.

To implement these principles effectively, it is essential to be able to evaluate ribosome loading efficiency, predict or measure both global and local 2D/3D structures, and optimize or generate mRNA sequences either in segments or as a whole. Since some principles may involve trade-offs (*e.g.*, optimizing codon usage might conflict with maintaining/increasing structural stability), the final mRNA sequence design must balance these competing objectives to achieve an optimal outcome.

## Optimizing and designing UTR sequence to enhance ribosome loading and translation efficiency

**Technical terms**

*Random Forest (RF)*: An ensemble learning method that constructs multiple decision trees during training and outputs the average prediction (for regression) or majority vote (for classification) of individual trees. Each tree is built using a random subset of the training data and features, which helps prevent overfitting.

*Multilayer Perceptron (MLP):* A basic type of feedforward artificial neural network that consists of an input layer, output layer, and one or more hidden layers with many neurons stacked together. Neurons in one layer are fully connected to every neuron in the next layer with nonlinear activation functions.

*Recurrent Neural Networks (RNN)*: A class of artificial neural networks designed to work with sequential data by maintaining an internal state (memory). This memory can capture information from previous inputs and update dynamically at each time step based on both current input and previous internal state. The temporal processing capability makes RNNs particularly effective for tasks involving sequences, such as natural language processing, speech recognition, and time series analysis.

*Convolutional Neural Networks (CNN)*: A type of artificial neural network specialized for processing grid-structured data, particularly images. CNNs use convolutional layers with learnable filters to automatically detect and extract hierarchical features from input data. They are particularly effective for computer vision tasks, such as image classification and object detection.

*Graph Neural Networks (GNN)*: A class of artificial neural networks designed to process and analyze graph-structured data, where nodes represent entities and edges capture relationships between them. By learning representations at multiple levels—nodes, edges, and entire graph structures—GNNs excel at tasks ranging from molecular property prediction in chemistry to social network analysis.

*Generative Adversarial Networks (GAN)*: A machine learning framework for generative artificial intelligence where two neural networks compete with each other in the form of a zero-sum game. A generator creates synthetic data, while a discriminator tries to distinguish between real and generated data. Through this competitive process, both networks improve simultaneously: the generator becomes increasingly adept at creating realistic data, while the discriminator becomes better at detecting subtle differences. This architecture has enabled breakthrough applications in image synthesis, art creation, and data augmentation.

*Autoencoders*: A type of artificial neural network that learns the lower-dimensional representation (encoding) of unlabeled data and then reconstructs it (decoding) from the encoding in an unsupervised fashion. The encoded representation often captures important features of the input data, which makes autoencoders suitable for various applications, such as dimensionality reduction, feature detection, and image generation.

*Long Short-Term Memory (LSTM) Networks*: A variant of RNNs that aims to mitigate the vanishing gradient problem commonly encountered by traditional RNNs. It uses specialized memory cells with input, output, and forget gates to control information flow. LSTMs are particularly good at learning long-term dependencies in sequential data compared to traditional RNNs.

*Gated Recurrent Unit (GRU)*: A simplified version of LSTM networks that uses update and reset gates to control information flow. GRUs help solve the vanishing gradient problem in standard RNNs while being computationally more efficient than LSTMs.

*Bidirectional Encoder Representations From Transformers (BERT)*: A transformer-based language model that learns to represent text as a sequence of vectors using self-supervised learning. BERT is pre-trained on large text corpora using masked language modeling and next-sentence prediction tasks, then fine-tuned for specific tasks. It is notable for its significant improvement over previous natural language processing models in capturing latent representations and complex contextual relationships in text.

*Large Language Model (LLM)*: A category of foundation models, typically transformer-based, trained on massive amounts of text data to understand and generate human-like text. LLMs can perform various language tasks like translation, summarization, and question-answering without task-specific training. They learn patterns and relationships in language through self-supervised learning on large-scale datasets.

### 5′ UTR

To maximize antigen protein expression in target host cells, artificially designed mRNA should feature a 5′ capped UTR with high ribosome loading efficiency. A straightforward approach for finding optimal 5′ UTR is to select 5′ UTR sequences from existing human mRNAs with known high expression efficiencies in the target cell type. As shown in Figure 2, multiple 5′ UTR candidates can be utilized for the designed mRNA vaccines, including those occurring naturally in various species. However, naturally derived 5′ UTRs may contain various regulatory elements that could also hinder ribosome loading under certain conditions (31, 42, 43, 44, 45). For instance, iron response element (IRE) can block ribosomal complex binding under low cellular iron levels (43) and the existence of upstream ORFs (uORFs) can reduce the translation efficiency by regulating the number of ribosomal preinitiation complexes (PICs) that enter ORF region (42). Therefore, only essential regulatory elements that positively promote ribosome loading would be retained when designing 5′ UTR sequences for mRNA vaccine. For instance, the HIV-1 5′ UTR contains over six functional structural motifs that regulate viral mRNA dimerization, splicing, gene expression, viral packaging, and replication. However, the different structural motifs may impact gene expression efficiency in mRNA vaccines in distinct ways. To identify and design the most efficient 5′UTRs to enhance mRNA vaccine’s gene expression efficiency remains a challenge. 5’ UTRs with essential components—such as the Human β-globin mRNA 5′ UTR, which consists of only two structured motifs—are widely employed in vaccine designs to optimize mRNA vaccines. In the case of mRNA-1273, a novel, designed, and patented 5′ UTR sequence (46), featuring a less complex structure, was utilized to achieve the desired vaccine performance. Further optimization can also be achieved through RNA base mutations or base-pair alterations in the 5′ UTR sequence, which can affect structural stability of the optimized mRNA. Additionally, more advanced approaches involve *de novo* design of the 5′ UTR sequence, where regulatory elements are manually selected and combined to achieve specific goals.

Statistics based on annotations from GENCODE v19 (47) indicate that only 30% of human 5′ UTRs are 100 nucleotides or shorter, with an average length of approximately 200 nucleotides (48). This vast sequence configurational space (*e.g.*, ∼ 4^200^) impedes efficient optimization or design of 5′ UTR sequences for mRNA vaccines. In recent years, various machine learning (ML) models have been proposed for modeling the effect of the 5′ UTR on protein expression, as well as its optimization and design. These models range from random forests (RF), convolutional neural networks (CNN), and generative adversarial networks (GAN) to language model (LM)-based approaches (48, 49, 50, 51, 52, 53, 54, 55, 56, 57). Generally, these models attempt to predict a variety of experimental quantities based on the given 5′ UTR sequences or their related biological features. Examples of these experimental quantities include mean ribosome load (MRL), a proxy for translation rate that measures the average number of ribosomes associated with a given RNA (49), and translation efficiency (TE), a ratio that quantifies the rate at which mRNA is translated into proteins (50). Studies often combine these models with various optimization algorithms to find specific 5′ UTR sequences that achieve improved translation efficiency. In the following, we briefly review recent ML approaches with a focus on their specific applications rather than technical details of the models.

To investigate the effect of different 5′ UTR sequence variants on translation, Sample *et al.* (49) trained a CNN based model using MRL values obtained from a massively parallel reporter assay (MPRA) based polysome profiling experiment. The experiment used a library of 280,000 synthetic gene sequences, each containing a 50-nt fully random sequence inserted between a defined upstream 25-nt sequence for polymerase chain reaction (PCR) amplification and a downstream enhanced green fluorescent protein (eGFP) coding sequence. The proposed CNN model, termed Optimus 5-Prime, consists of three convolutional layers and a fully connected layer. It takes one-hot^2^ encoded 5′ UTR sequences as input and predicts their MRLs. Trained on 260,000 sequences, the model could explain 93% of the MRL variation (measured by the square of the Pearson correlation coefficient) in the test set with 20,000 sequences, a significant improvement over various position-specific k-mer linear models (49). The same experiment was also performed on a synthetic 5′ UTR library derived from ClinVar database (58) that has approximately 25,000 UTRs consisting of the first 50 nucleotides upstream of the start codon of 22,747 common and 2253 variant human 5′ UTR sequences. The model could explain 82% of the observed MRL variation and identify the effect of various variants on ribosome loading. Further evaluation showed the model’s ability to predict MRL for modified mRNAs containing pseudouridine/1-methyl pseudouridine, albeit with lower performance. Coupled with a genetic algorithm, the authors also demonstrated the model’s ability to design new 5′ UTRs targeting specific levels of protein expression. A major drawback of the model is the lack of flexibility in modeling 5′ UTRs of varying length, as the fixed-length input requires longer sequences to be truncated, resulting in information loss (48).

As the majority (∼70%) of human 5′ UTRs are longer than 100 nt (48), Optimus 5-Prime, trained with a fixed-length input (*e.g*., 100-nt), cannot generalize well to longer sequences. To alleviate this issue, Karollus *et al.* (48) proposed a similar CNN model, termed FramePool, that extends the capabilities of Optimus 5-Prime to handle 5′ UTRs of any length. The key idea behind FramePool is translation frame-dependent pooling, where convolutional outputs are separated according to the underlying biological reading frame, and global pooling is performed for each frame separately. When trained and tested on the same dataset of 280,000 sequences containing 50-nt random synthetic sequence in the 5′ UTR, both Optimus 5-Prime and FramePool exhibited similar performance in predicting MRL values. However, when applying both trained models to a test set consisting of 7600 sequences with lengths ranging from 25 to 100 nt (49), the Pearson correlation coefficient dropped to 0.743 for Optimus 5-Prime while achieving 0.901 for FramePool. This shows that the frame pooling technique enables the model to treat sequences of variable length and allows for effective generalization to 5′ UTR sequences considerably longer than those used in training. Nevertheless, the FramePool-predicted MRLs showed considerably lower correlation (ranging from 0.11 to 0.25 for Pearson correlation coefficient) with the experimentally measured TEs when tested on six datasets containing human transcripts (59, 60, 61, 62, 63, 64). This is possibly due to the fact that both the CDS and the 3′ UTR, which have been shown to affect the ribosome load and protein-to-mRNA ratios (PTR) (63, 64, 65), vary between transcripts in endogenous data. It was also found that FramePool-predicted effects on MRL for single nucleotide variants (SNVs) correlated with the level of phylogenetic conservation at that position, a trend especially pronounced for nucleotides within the 100 nt of the canonical start codon. Furthermore, integrating the models with the Kipoi framework (66), an API and repository of ready-to-use trained models for genomics, enables practitioners to efficiently analyze any human 5′ UTR variant or mutation, including indels.

Cao *et al.* (50) developed a high-throughput strategy to design, screen, and optimize 5′ UTRs for enhanced protein expression. By training a random forest (RF) model on a dataset of naturally occurring 5′ UTRs with high translation efficiencies, the authors created a total of ∼12,000 100-bp 5′ UTR libraries containing 3586 synthetic sequences (generated using a genetic algorithm) and 8414 natural sequences. The library was subsequently screened using a recombinase-mediated integration strategy. The RF model was developed to predict TE based on 5′ UTR characteristics, such as k-mer frequency, RNA folding energy, 5′ UTR length, and number of ORFs. Further validation of the top-predicted 5′ UTR hits that increased green fluorescent protein (GFP) production in HEK 293T cells enabled the authors to identify three synthetic 5′ UTRs that outperformed commonly used non-viral gene therapy plasmids in expressing protein payloads. This combination of experimental and computational techniques provides a robust strategy for the systematic discovery and engineering of 5′ UTRs to enhance protein expression.

Naturally occurring mRNA sequences, unlike synthetic ones, contain differing coding sequences and 3′ UTRs that can also affect translation regulation (63, 64, 65). Thus, the sequence motifs learned by existing ML approaches trained on a single dataset (single-task models) may not generalize well to other datasets (48, 55, 56). Instead of relying on single-task models, Zheng *et al.* (55, 56) proposed MTtrans, a multi-task learning model that integrates information from multiple datasets. The key component within MTtrans is the shared CNN layers, where task-specific inputs are transformed to task-specific feature maps. The feature maps are subsequently fed into task-specific network layers to predict related experimental quantities for the corresponding tasks. By using the same CNN layers across different tasks, MTtrans is forced to learn the shared patterns from multiple experimental systems. This approach is based on the fundamental assumption that the 5′ UTR sequence features extracted by shared CNN layers that can robustly predict translation rate across multiple datasets are more likely to be actual underlying regulatory elements governing mRNA translation. The results show that MTtrans outperforms both Optimus 5-Prime and FramePool for MRL prediction on the same MPRA datasets with synthetic 5′ UTRs and naturally occurring endogenous human 5′ UTRs. Using fluorescence-activated cell sorting coupled with a deep sequencing (FACS-seq) experiment, the authors further validated the impact of most motifs identified by the learned shared CNN encoder. These results indicate that sequence features identified through multi-task learning are generalizable across different experimental systems, highlighting its strength in identifying evolutionarily conserved sequence motifs.

To facilitate the generation of 5′ UTR sequences, Barazandeh *et al.* (54) proposed UTRGAN, a generative adversarial network (GAN) coupled with an optimization procedure to generate 5′ UTR sequences with desired features such as high ribosome load and TE, while still mimicking various properties of natural UTR sequences. Both UTRGAN’s generator and discriminator are based on convolutional neural networks, where the generator learns to generate plausible sequences from samples drawn from a latent space (low-dimensional space containing compressed representations of the one-hot encoded input sequences), while the discriminator learns to distinguish the decoy data from the true ones. These two models are trained adversarially together in a zero-sum game until the discriminator model is no longer able to differentiate generated data from real ones, meaning the generator model is generating plausible examples. The results showed that the UTRGAN-generated 5′ UTRs maintain similar 4-mer, GC content, predicted MRL, TE, and minimum free energy (MFE) distributions compared to natural ones. The optimization pipeline in UTRGAN employs an iterative procedure where, in each iteration, the generated 5′ UTRs are evaluated by off-the-shelf ML prediction models, such as FramePool (48) and MTtrans (55, 56), to guide the direction of updates in the latent space through a gradient ascent algorithm.

More recently, several RNA language models (LMs) have emerged and exhibited better performance on 5′ UTR function-related prediction tasks, examples include RNA-FM (52) and UTR-LM (57). The key component behind these LMs is pre-training on large sequence datasets, which enables the extraction of meaningful semantic representations from raw RNA sequences. Compared to multi-task learning employed in MTtrans, the self- or semi-supervised nature of pre-training allows LMs to take advantage of massive amounts of unlabeled RNA sequence data, avoid solely relying on labeled information, and extract transferable meaningful semantic representations of RNA sequences across various biological systems. RNA-FM, proposed by Chen *et al.* (52), is an RNA foundation model based on the bidirectional encoder representations from transformers (BERT) language model architecture. It is built upon 12 transformer-based bidirectional encoder blocks and trained on 23 million sequences from the RNAcentral database (67) in a self-supervised manner. With simple CNN and multilayer perceptron (MLP) based prediction modules, RNA-FM can benefit various downstream tasks, including RNA secondary structure prediction, RNA 3D modeling, RNA-protein interaction modeling, and mRNA gene expression regulation modeling. Specifically, when trained on the same synthetic dataset containing 76,319 distinct random 5′ UTRs with lengths spanning from 25 to 100 nucleotides (49), the RNA-FM-based model achieves better correlations in MRL predictions than Optimus 5-Prime on two test sets consisting of 7600 random and 7600 human 5′ UTRs with varying lengths, respectively. UTR-LM, proposed by Chu *et al.* (57), is also a transformer-based model pre-trained to extract representations from the raw sequences *via* nucleotide masking and reconstruction. Compared to the RNA-FM, the initial input feature utilized in the pre-training stage not only contains 1D sequence information but is also further augmented with supervised information including predicted secondary structure and predicted MFE. The pre-trained model was fine-tuned on a variety of downstream tasks, outperforming Optimus, FramPool, MTtrans, Cao-RF, and RNA-FM on MRL and TE prediction in terms of Spearman’s rank correlation coefficient. Additionally, the authors demonstrated UTR-LM’s ability to design high-efficiency 5′ UTRs; specifically, 211 new 5′ UTRs with high predicted TE values were tested experimentally, and the results confirmed that the top-designed sequence achieved a 32.5% increase in protein production level relative to well-established 5′ UTRs optimized for therapeutics.

These recent studies demonstrate that ML models can effectively learn the complex sequence-function relationships of 5′ UTRs from large datasets, providing both insights into the regulatory code as well as enabling the rational design of optimized 5′ UTR sequences for applications requiring precise control of protein expression levels. However, it remains crucial to understand the structural characteristics of these designed or optimized sequences. This understanding ensures that specific 2D/3D structures are formed and/or conserved sequences are appropriately exposed for the proper function of desired regulatory elements. Given that 5′ UTRs from human genes average ∼218 nucleotides in length (38), experimentally determining the 3D structure of the entire sequence can be challenging. Nevertheless, other experimental techniques, such as chemical probing (68, 69, 70, 71), can provide RNA secondary structural information at single-nucleotide resolution and guide 2D structure prediction (72). Additionally, various computational tools can also offer valuable insights for analyzing the 2D/3D structures of 5′ UTRs (73, 74, 75, 76). Interestingly, a recent study indicates that the structure of mature mRNAs correlates significantly with the structure of the corresponding cotranscriptionally folded pre-mRNAs (77). Therefore, when using various computational models to predict 2D/3D structures for designed 5′ UTRs, incorporating additional kinetic folding or cotranscriptional folding information might be useful.

### 3′ UTR and poly(A) tail

While not as critical as the 5′ UTR in regulating mRNA expression, the 3′ UTR contains several key regulatory elements that can still significantly impact overall protein expression by influencing mRNA lifetime within the cell. For instance, AU-rich elements (AREs)—usually 50 to 150-nucleotide sequences containing multiple AUUUA motifs—promote mRNA decay through interactions with ARE-binding proteins (31). Another important element is the microRNA (miRNA) response elements (MREs). When bound by miRNAs, it can lead to translational repression, preventing the mRNA from being translated into protein (78, 79, 80). Of utmost importance is the poly(A) tail, which contains binding sites for PABPs. This crucial element not only protects mRNA from Ribonucleases (RNases) but also facilitates ribosome loading through eukaryotic initiation factors (eIFs)-mediated end-to-end interactions during mRNA circularization (44, 81, 82). Although many miRNA and mRNA targets remain unknown, computational and experimental tools can be employed to identify potential miRNA targets (83, 84, 85, 86, 87). In principle, negative regulators such as MREs are excluded when designing 3′ UTRs, and one can utilize certain human mRNAs with 3′ UTRs devoid of MREs. However, excluding MREs could interfere with other essential regulatory elements within the 3′ UTRs that may either promote or suppress protein translation. Additionally, available human mRNA 3′ UTRs lacking MREs may not be optimal for efficient protein translation. All these factors should be carefully considered and optimized.

The human β-globin (hGb) 3′ UTR is widely used in IVT mRNA design due to its high effectiveness in enhancing protein expression (88). Recent studies have shown that other naturally occurring 3′ UTR sequences can match or even surpass the performance of the hGb 3′ UTR in this regard (89, 90). Currently, experimental approaches such as the systematic evolution of ligands by exponential enrichment (SELEX) remain crucial for optimizing 3′ UTR sequences. SELEX experiments have successfully identified 3′ UTR sequences that can significantly improve protein expression (91), with some enhancing total protein expression by over threefold across various coded proteins. Unlike the extensive computational efforts in 5′ UTRs design and optimization, 3′ UTRs have not yet benefited from computational modeling due to the lack of a comprehensive database for training and validation. Nevertheless, various structure prediction models can still provide valuable insights into 3′ UTR structures, helping to estimate their interactions with various 3′ UTR-binding factors (*e.g.*, ARE-binding proteins and MRE-binding miRNAs).

## Optimizing ORF codon to improve translation efficiency

The ORF encodes the sequential information of a protein’s amino acids. Due to codon degeneracy—where multiple distinct three-base pair codons can encode the same amino acid—one of the crucial aspects of ORF design involves finding a proper codon combination that matches the desired amino acid sequence and optimizes protein synthesis at the same time.

Codon optimization is the process of selecting an ideal codon for each amino acid. A common strategy aims to enhance translation rates by replacing rare codons with synonymous ones favored by the cellular environment, guided by the abundance of corresponding transfer RNAs (tRNAs) in host cells. This artificial synonymous codon usage bias can be quantified using the codon adaptation index (CAI) (92), a useful metric for estimating a gene’s expression level. For a given ORF encoding a specific amino acid sequence, the CAI is calculated as the geometric mean of the relative frequencies of all codons used. The relative codon frequency of a particular codon is defined as its observed frequency divided by the frequency of its corresponding most prevalent synonymous codon within a reference set of highly expressed genes. Using the most prevalent codons for all amino acids in an ORF results in a CAI of 1.0. Notably, approved mRNA vaccines, such as BNT-162b2 and mRNA-1273, have optimized ORFs with CAI values exceeding 0.9 (23). Additionally, multiple experiments have demonstrated that codon selection can significantly influence the intracellular lifespan of mRNA (93, 94, 95, 96, 97, 98, 99, 100). Therefore, the codon stabilization coefficient (CSC), derived from the correlation between codon frequencies and mRNA half-lives, can serve as another quantitative metric for codon optimization (93).

The elongation of the amino acid chain requires the locally structured ORF sequence to partially unfold into a single-stranded form. This unfolding allows the ribosomal complex to move towards the 3′ end of the mRNA. However, the local structure of the ORF should not be so stable that it resists unfolding, as this could block amino acid chain elongation at that position (92). A model proposed by Paulo Gaspar *et al.* for optimizing mRNA ORF 2D structures employs a pseudo-minimum free energy (pseudo-MFE) based algorithm (101). This approach estimates the MFE of a given mRNA sequence by averaging the interaction energies of all possible single stem-loop conformations. Unlike methods that enumerate every possible 2D structure for a given sequence, this algorithm significantly reduces computational time, enabling the optimization of mRNA sequences exceeding 1000 codons. The goal of this model is to maximize the MFE for the sequence, thereby avoiding local stable structures that could potentially reduce translation efficiency.

## Refining global sequence to improve structural stability

RNA, unlike DNA, is prone to base-catalyzed hydrolysis due to the 2′ hydroxyl group on its ribose sugar. This hydrolysis can occur spontaneously in solution, even without catalysts or enzymes, and is more likely in single-stranded regions where sensitive chemical groups are exposed (102). To maintain vaccine efficacy, mRNA vaccines must be stored at very low temperatures to minimize natural degradation (102). mRNA with well-structured regions, fewer single-stranded motifs, and higher GC content tends to have lower degradation rates, potentially allowing for more flexible storage requirements (103, 104, 105). The folded structures of ORFs can vary significantly based on codon selection, leading to substantial differences in structural stability among ORFs with different sequences. Therefore, a key principle in ORF design is prioritizing stable structures by identifying sequences that yield folded structures with lower MFE, thereby enhancing overall mRNA stability (104).

In the past, several computational tools have been developed to identify sequences capable of folding into low-energy structures at the 2D level. CDSfold, a model developed by Goro Terai *et al.*, aims to identify sequences with stable MFE structures (106). CDSfold searches for the ORF sequence that can be translated into the desired protein while simultaneously forming the most stable 2D structure by employing dynamic programming (107), a computationally efficient method to determine the MFE and its corresponding RNA structure. However, with the time complexity of *O(L*^*3*^*)* and space complexity of *O(L*^*2*^*)*, where *L* represents the sequence length, the model may take several hours to analyze an mRNA ORF consisting of just a few thousand codons.

LinearDesign, a recently developed model, addresses the computational complexity inherent in sequence optimization tasks by employing a classical lattice parsing approach from computational linguistics (108). This innovative method draws an analogy between identifying the optimal mRNA sequence and selecting the most coherent sentence among similar-sounding alternatives in linguistics. By leveraging an enhanced left-to-right dynamic programming algorithm within the framework of lattice parsing, LinearDesign significantly reduces the time required to identify the mRNA sequence folding to the stable MFE structure (109). This advancement cuts processing time from several hours to minutes, marking a substantial improvement in computational efficiency. When tested for designing mRNA vaccines for SARS-CoV-2, LinearDesign successfully identified mRNA sequences capable of achieving over threefold higher protein expression in cells compared to established vaccines like BNT162b2 and mRNA-1273 (108). Additionally, it significantly increased antibody titers by up to 128 times in mice compared to the codon-optimization benchmark for mRNA vaccines targeting SARS-CoV-2 and varicella-zoster virus (VZV).

Predicting RNA structures from sequences is crucial for assessing and understanding structural stability. Most efforts in this field remain focused on 2D structures. For traditional (non-ML-based) RNA structure prediction methods, thermodynamic parameters and force fields are critical for accurately predicting both 2D and 3D RNA structures. For instance, in RNA 2D structure prediction, the nearest-neighbor free-energy model and Turner parameters are widely used (110, 111), and employed by models such as RNAfold (112), RNAstructure (72), mfold (113), and Vfold2D (114, 115, 116). Turner parameters represent a set of nearest-neighbor thermodynamic parameters for RNA folding, derived from experimental measurements. These parameters provide free energy and enthalpy/entropy changes for forming various RNA secondary structure motifs and are widely used in thermodynamics-based RNA secondary structure predictions. Existing thermodynamic-based RNA 2D structure prediction models face challenges for RNAs containing complex structural elements such as multibranched junction loops and pseudoknots (73). These limitations stem from the lack of accurate thermodynamic parameters for these elements. In practice, it is often useful to consider the consensus base pairs predicted from different models.

In addition to traditional folding energy, an alternative metric for assessing mRNA structural stability is the average unpaired probability (AUP), which reflects the overall ‘unstructuredness’ of the RNA (104). In principle, AUP may provide more relevant information about mRNA in-solution lifetime at a given storage temperature, because mRNA degradation may correlate with the total number of unpaired nucleotides (104). Wayment-Steele *et al.* developed the RiboTree model to optimize mRNA ORFs to achieve the lowest AUP (104). RiboTree utilizes a Monte Carlo tree search algorithm for stochastic minimization and the LinearPartition algorithm to compute the AUP for designed mRNA sequences (104, 109). The model successfully identified ORF sequences that can enhance the in-solution lifetime of COVID-19 mRNA by more than twofold in prediction.

To theoretically assess mRNA in-solution stability and minimize hydrolytic degradation of designed mRNA sequences, Leppek *et al.* proposed a linear regression model called DegScore (22) that can estimate mRNA in-solution degradation rate. Trained on a large-scale in-line chemical probing dataset comprising 3030 RNA fragments, DegScore can quantitatively capture properties related to mRNA degradation and predict mRNA in-solution lifetime (22). While ORF sequences corresponding to the lowest AUP, DegScore, or MFE are generally different, these metrics have been experimentally validated as useful for enhancing mRNA vaccine stability, both in solution and within cells (22).

ML-based models for RNA structure prediction have recently emerged, demonstrating significant capabilities in predicting 2D or 3D structures from sequences (73, 117). Several models have been developed and evaluated for designing short ORF sequences (102–130 nucleotides) in competitions like OpenVaccine (https://eternagame.org/challenges/10845741). By leveraging various architectures, such as autoencoders, CNNs, graph neural networks (GNNs), gated recurrent units (GRUs), and long short-term memory (LSTM) networks, these models have enhanced the prediction of mRNA degradation (118, 119, 120, 121, 122, 123). However, ML-based models also face some limitations. They have not yet surpassed traditional RNA structure prediction models in terms of prediction accuracy, particularly for artificially designed RNAs, as demonstrated in the 15th critical assessment of structure prediction (CASP15) competition (73, 75). Additionally, current ML methods have not yet been extensively evaluated for large RNAs, especially for mRNAs containing thousands of codons.

## Fine-tuning local sequence to extend the in-cell lifetime and increase expression fidelity

In addition to the global structure of ORFs, local structural elements such as loops, hairpins, junctions, and pseudoknots can significantly influence mRNA in-cell lifetime (124), local translation rate, and translation fidelity (125). These RNA local structures, formed within short sequence regions, play a crucial role in regulating mRNA in-cell degradation (124), ribosome moving rate (92), and potential translational frameshift stimulation (126).

mRNA can be quite unstable in cells due to the RNases-dependent RNA degradation (102, 127, 128, 129). The cellular RNase system includes two main types of enzymes: exoribonucleases (130, 131, 132)—enzymes that degrade RNA by removing terminal nucleotides from either the 5′ ends or the 3′ ends of the RNA molecule, and endoribonucleases (133, 134)—enzymes that cleave either single-stranded or double-stranded RNA chain by recognizing specific RNA sequence and structural motif (102, 129). To suppress endoribonuclease activation, several specific RNA sequences or structure elements should be avoided. Since single-stranded RNA (*i.e.* loops) is a common structural element recognized by various types of endoribonucleases (102, 129), designing mRNA sequences with fewer single-stranded regions is a practical way to increase mRNA in-cell lifetime. This goal aligns well with the optimization objective (lowest AUP) employed in the RiboTree model (104).

An interesting finding from the benchmark test of LinearDesign is that in-solution structural stability, indicated by the folding free energy, shows only a weak correlation with in-cell lifetime and protein expression for a typical mRNA sequence (108). This may be attributed to the optimization of the mRNA ORF for higher stability, which can hinder decoding progression due to ribosomal stalling caused by rare codon usage or the formation of stable local secondary structures (135, 136, 137). Ribosomal stalling during mRNA translation can result in ribosome collisions at specific sites, ultimately leading to mRNA cleavage and degradation (138, 139, 140). Such nonsense-mediated RNA decay significantly shortens the in-cell lifetime of mRNA and reduces protein expression efficiency (138, 139, 140, 141). These mechanisms suggest the complexity of the secondary structure and folding stability-based ORF design for mRNA vaccines.

Protein cotranslational folding (142, 143), similar to RNA cotranscriptional folding, is a kinetic process highly sensitive to the elongation rate of the amino acid chain (142). Wild-type mRNA for an antigen typically incorporates specific codon sequences and structural elements within the ORF to regulate the elongation rate, ensuring the nascent amino acid chain folds into functional structures (142, 144, 145). In artificially designed mRNA for vaccines, however, the ORF sequence may be altered due to codon optimization. Consequently, the programmed elongation rate of the amino acid chain during translation may differ (125), potentially leading to the production of misfolded proteins (146). Structural misfolding can result in significant differences between the expressed protein and the target antigen. In more severe cases, such structural alterations may render the protein toxic to cells (*e.g.*, misfolded Aβ protein associated with Alzheimer’s disease (147)). Several computational tools are available to predict protein cotranslational folding while accounting for translation rates (143, 146). These models can aid in codon optimization by identifying and avoiding sequences likely to result in misfolded structures.

Programmed translational frameshift, observed in many viruses and eukaryotes, requires specific RNA sequences and locally folded structures such as stem-loops or pseudoknots (126). Designed ORF sequences may inadvertently contain these sequence/structural elements, potentially stimulating unintended translational frameshifts (148, 149). This can lead to the expression of alternative proteins, increasing the risk of side effects. COVID-19 mRNA vaccines have been reported to produce undesired proteins in clinical applications (150). Studies have also shown that up to 30% of expressed proteins in host cells have altered amino acid sequences due to translational frameshifts (150, 151).

However, current ORF sequence design tools cannot explicitly consider local structures. One potential approach is to predict global structures using various state-of-the-art models, including physics-based and machine learning-based models, and then search for consensus local structural elements within the predictions. Table 1 provides a summary of selected computational tools for RNA structure prediction. An alternative approach involves computing base pairing probabilities from the partition function and subsequently inferring local structures from these probabilities (152, 153). Identifying local structures can help pinpoint regions susceptible to problematic conformations, which may adversely impact translation efficiency and fidelity.

**Table 1:** RNA structure prediction tools

2D structure prediction		3D structure prediction	
Model	Method	Model	Method
RNAstructure (72)	TM	iFold (184)	MD
RNAfold (112)	TM	SimRNA (185)	MC
RNAalifold (186)	TM	RNAComposer (187)	AS
PKNOTS (188)	TM	FARNA (189)	MC
Mfold (113)	TM	MC-Sym (190)	AS
Vfold2D (114, 115, 116)	TM	FARFAR2 (191)	AS
HotKnots (192, 193)	TM	ARES (194)	ML
CentroidFold (195)	TM	IsRNA (196, 197)	CGMD
IPknot (198)	TM	RNAJP (199)	MD/MC
LinearFold (200)	TM	RNA3DCNN (201)	ML
TurboFold (202)	TM	PaxNet (203)	ML
SPOT-RNA (204)	ML	DeepFoldRNA (205)	ML
E2Efold (206)	ML	trRosettaRNA (207)	ML
MXfold2 (208)	ML	epRNA (209)	ML
EternaFold (210)	ML	E2Efold-3D (211)	ML
Ufold (212)	ML	ReseTTAFoldNA (213)	ML
DMfold (214)	ML	DRFold (215)	ML
CNNfold (216)	ML	AlphaFold3 (217)	ML
RNAformer (218)	ML	3dRNA (219)	AS
2dRNA (220)	ML	Vfold-Pipeline (221)	AS

AS, Assembly; CGMD, Coarse-Grained Molecule Dynamic; MC, Monte Carlo; MD, Molecule Dynamic; ML, Machine Learning; TM, Thermodynamic.

## Multi-objective optimization

The sequence design of mRNA vaccines generally involves multiple objectives. Given the intrinsic properties of mRNA molecules, achieving all objectives within a single sequence can be challenging. For example, maximizing structural stability often conflicts with the optimal codon selection.

Recent studies on ORF design have demonstrated that optimal antigen expression cannot be achieved by simply optimizing CAI, MFE, or AUP alone. Instead, the best-performing ORF sequences typically find a balance between CAI and structural stability (22). Based on this finding, several approaches have been proposed to optimize both translation efficiency and mRNA stability within the cell, thereby achieving optimal protein expression efficiency for designed vaccines. These models utilize a combined scoring function that integrates CAI with MFE or AUP. For example, CDSfold uses $\text{MFE}\times {\text{CAI}}^{\lambda }$ to combine the two factors, while LinearDesign uses MFE−λlog(CAI). However, determining the optimal parameter λ in these scores is nontrivial, as benchmark experiments have shown that the best-performing ORF sequences correspond to different λ values for different protein targets of SARS-CoV-2 and VZV (108). Rather than relying on a single value, LinearDesign employs a range of λ to explore a broader region of candidate sequences and evaluates all candidates through additional experiments (108). This approach enables exploring previously unreachable yet important sequence space.

**Figure 3:**
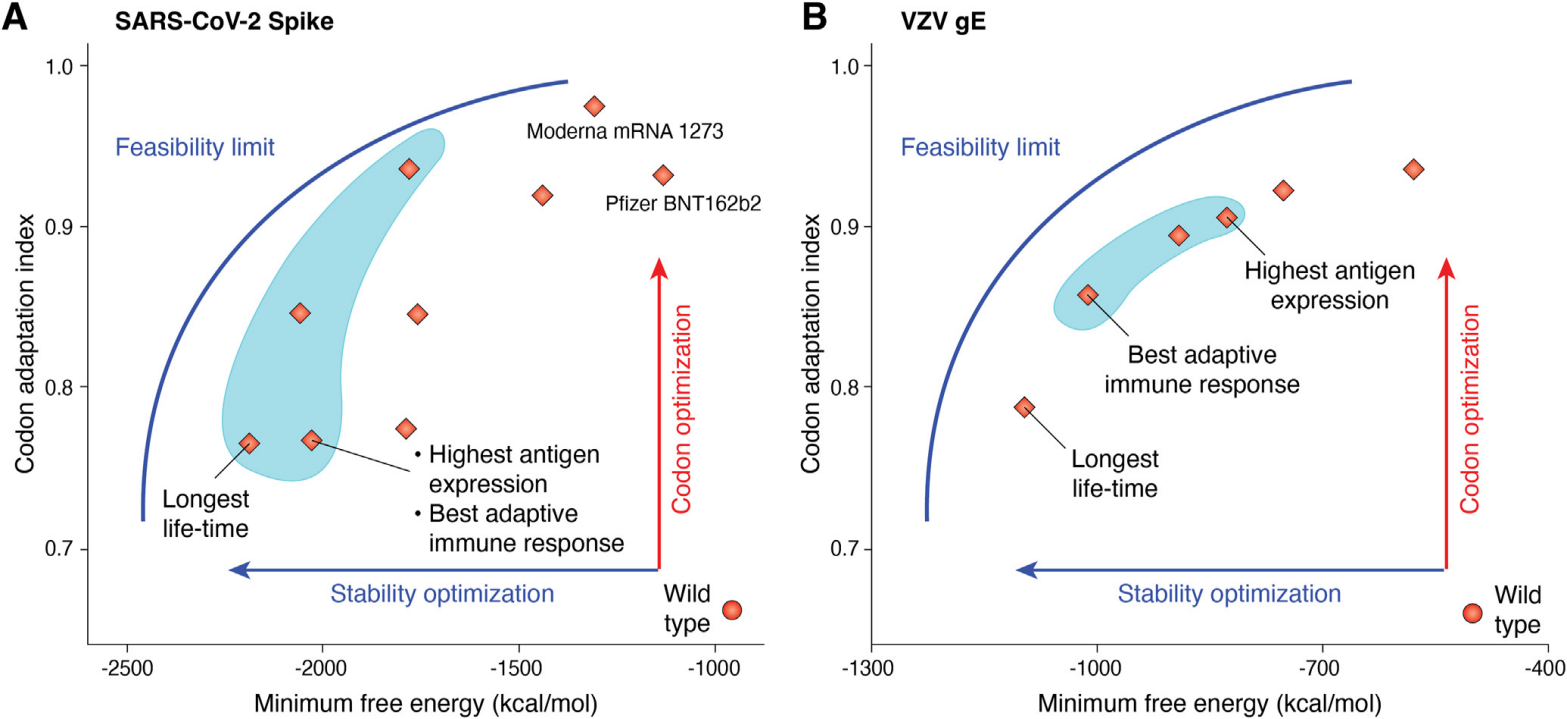
**Computationally designed mRNA vaccines.** Sequences for (*A*) severe acute respiratory syndrome coronavirus 2 (SARS-CoV-2) and (*B*) varicella zoster virus (VZV), together with their calculated codon adaptation indices (CAIs), minimum free energies (MFEs), and various experimentally measured properties. In both (*A*) and (*B*), the areas to the right of the feasibility limit curves represent possible regions for designing mRNA sequences. Light blue shaded areas indicate potential high-efficiency mRNA vaccine regions predicted by LinearDesign (108). For mRNA vaccines that encode either SARS-CoV-2 spike protein or VZV gE protein, the best-performing one (*i.e.* inducing the strongest adaptive immune response) has neither the highest CAI nor the lowest MFE. Data is obtained from reference (108).

As shown in Figure 3 for the computationally designed mRNA vaccines for SARS-CoV-2 and VZV, identifying the optimal ORF sequence requires balancing multiple factors beyond CAI and structural stability. Codon selection, local structural elements, and various other factors must be considered concurrently. Although LinearDesign can identify potential high-efficiency regions for mRNA sequence design (see Fig. 3), determining the exact “best” sequence among the suggested candidates remains a challenge. The integration of different mRNA components (*e.g.*, UTRs and ORF) introduces additional challenges, such as long-range interactions between ORFs and UTRs, and effects induced by trans-activating regulators within the UTRs.

Recent advancements in deep learning-based techniques have demonstrated significant advantages in multi-objective optimizations (154, 155). As discussed in the section *Optimizing and designing UTR sequence to enhance ribosome loading and translation efficiency*, several approaches have been developed to optimize 5′ UTRs by considering multiple objectives. Nevertheless, applying deep learning methods to ORF design remains challenging due to the scarcity of existing databases. To build comprehensive databases, mRNA ribosome load, in-solution stability, in-cell lifetime, and translation efficiency need to be experimentally measured across a broad range of diverse sequences. Addressing this need, Leppek *et al.* developed PERSIST-seq, an RNA sequencing-based platform that systematically delineates key properties of designed mRNAs (22). Currently, 233 mRNA vaccine sequences, along with their associated key properties, have been deposited through the application of PERSIST-seq (22). Deep learning models developed to predict gene expression (156, 157) may be extended to IVT mRNA as available databases rapidly expand. Furthermore, given the remarkable capabilities of generative artificial intelligence (GAI) in producing various types of biological data (158, 159, 160), transformer-based deep neural networks, such as those used in large language models (LLMs), show great potential for designing novel mRNA vaccine sequences.

## Utilizing RNA modifications to improve mRNA vaccine efficacy

Hundreds of natural modifications have been identified and characterized for RNAs. These modifications play a critical role in regulating mRNA functions (161, 162, 163, 164, 165, 166), ranging from reducing mRNA immunogenicity (163, 164) and enhancing mRNA stability (162) to modulating mRNA translation (163, 164). RNA modifications have been employed in vaccine design and clinical and preclinical mRNA therapeutic applications (164). A notable application is in SARS-CoV-2 mRNA vaccines, such as Moderna’s mRNA-1273 and BioNTech/Pfizer’s BNT-162b2, where the modified nucleobase N1-methyl pseudouridine (m1Ψ) has proven crucial for vaccine efficacy (163, 167). Another widely utilized modification in mRNA therapeutics is pseudouridine (Ψ), which can significantly enhance mRNA translation efficiency (168, 169). Beyond SARS-CoV-2 mRNA vaccines, other examples include vaccines targeting influenza viruses (170), cytomegalovirus (CMV) (171), HIV (172), Ebola (173), Zika (174), and human metapneumovirus (hMPV) (175), *etc.* Here, we focus on the impact of nucleoside modifications in 5′ UTR, ORF, and 3′ UTR regions on mRNA vaccine efficacy from a structural perspective. For a comprehensive overview of how other modifications—such as those in 5′-cap, backbone, and poly(A) tail modifications, as well as the enzymatic ligation of nuclease-resistant oligonucleotides to the poly(A) tail (176)—affect mRNA efficacy, readers are directed to additional reviews (164, 165).

From a structural perspective, RNA modifications can influence RNA folding by altering the structural and energetic properties of base pairing and stacking, including conformational flexibility, groove hydrophobicity, and the stability of long-range contacts (177, 178, 179). As a result, these modified bases could potentially alter RNA secondary structure. For instance, a chemical probing study suggested that RNAs containing m1Ψ and uridine may adopt distinct secondary structures (128). Additionally, optical melting experiments on synthetic short RNA duplexes indicated that Ψ and m1Ψ modifications, compared with the unmodified uridine (U), could increase the stability by 0.25 and 0.18 kcal/mol, respectively, for each base pair (128). However, studies have also indicated that m1Ψ can reduce translational fidelity by causing +1 ribosomal frameshifting, producing altered proteins (150, 151). Therefore, various aspects must be considered when designing predictive computational models.

To predict the impact of modified nucleotides in RNA structure and stability, the ViennaRNA package includes six modified nucleotides such as Ψ by incorporating energy corrections derived from experimental data (180, 181), and RNAstructure employs linear regression to fit thermodynamic parameters for modified nucleotides such as N6-methyladenosine (m6A) (182). Integrating modified bases in RNA structure prediction remains an ongoing endeavor. With the expanding experimental data on the thermodynamics of modified nucleotides, we anticipate that more accurate models will emerge to facilitate the design and optimization of mRNA vaccine sequences and structures.

## Summary and perspective

The rapid advancement of mRNA vaccine technology has provided an increasingly promising tool against infectious diseases and cancers. While mRNA vaccines have demonstrated significant success in preventing COVID-19, their application in other areas remains limited. Various computational models, including advanced ML approaches trained on large experimental 5′ UTR datasets, now enable the evaluation, optimization, and rational design of mRNA sequences for diverse applications. However, the scarcity of data poses a major obstacle in applyingpowerful ML models to mRNA vaccine design. Additionally, the limited accuracy and scalability of the RNA 2D/3D structure prediction tools —especially for sequences containing thousands of nucleotides—further hinder the design process. Looking forward, several areas warrant further research and development.

1. Integrated sequence design/optimization: while current approaches focus on optimizing individual mRNA components, future research should aim to develop integrated models that consider both the global mRNA structure which determines in-solution stability and the local structure/sequence motif which affects the mRNA translation efficiency, translation fidelity, and in-cell degradation rate. These models should also consider alternative folding pathways, such as cotranscriptional folding, and intermediate (subpopulated) states, which can lead to structures different from the thermodynamic equilibrium MFE structure.
2. Expanding current mRNA vaccine-related databases: The expansion will likely require extensive experimental measurements of various properties for mRNA sequences, such as translation efficiency, translation fidelity, in-solution stability, in-cell lifetime, and reactogenicity. Additionally, experimental thermodynamic parameters for modified nucleotides can further benefit various RNA 2D/3D structure prediction tools (see Table 1).
3. Balancing multiple objectives: Current mRNA vaccine design principles often focus on individual objectives. Future research should prioritize developing optimization algorithms that can effectively balance competing design goals to identify a globally optimal mRNA sequence. Achieving this will require a deep understanding of the underlying mechanisms driving the immune response triggered by mRNA vaccines.
4. Experimental validation: while computational approaches offer powerful tools for mRNA vaccine design, rigorous experimental validation remains essential. Developing high-throughput screening methods to test computationally designed sequences will be crucial for advancing the field.

In conclusion, computational approaches hold tremendous potential for advancing mRNA vaccine design and optimization. By integrating biological knowledge, machine learning techniques, and experimental validation, these methods can greatly improve the efficacy, stability, and safety of mRNA vaccines for emerging infectious diseases and therapeutic areas such as cancer immunotherapy.

## Data availability

The data supporting the findings of this study are available in the manuscript.

## Declaration of generative AI in scientific writing

The large language model (LLM), Claude, developed by Anthropic was used for language refinement only.

## Conflicts of interest

The authors declare that they have no conflicts of interest with the contents of this article.

## Footnotes

1 Reactogenicity refers to the ability of a vaccine to elicit expected shortterm adverse reactions or inflammatory responses following immunization. Reactogenic events can include local effects, such as pain, redness, swelling, or induration at the injection site, as well as systemic effects like myalgia, fever, and headache. Ideally, vaccines should induce a robust immune response while minimizing reactogenicity.

## Footnotes

2 One-hot encoding is a technique that represents categorical variables as binary vectors. Each unique category is mapped to a specific position in the binary vector, where a value of one indicates its presence and a value of 0 indicates its absence.
